# Prevention of Diabetic Complications by Activation of Nrf2: Diabetic Cardiomyopathy and Nephropathy

**DOI:** 10.1155/2012/216512

**Published:** 2012-05-08

**Authors:** Bing Li, Shujun Liu, Lining Miao, Lu Cai

**Affiliations:** ^1^Department of Nephrology, Second Hospital of Jilin University, Changchun 130042, China; ^2^Department of Nephrology, Jilin Province People's Hospital, Changchun 130041, China; ^3^KCHRI Pediatric Diabetes Research Laboratories, Department of Pediatrics, The University of Louisville, Baxter I, Suite 304F, Louisville, KY 40202, USA

## Abstract

Diabetic cardiomyopathy and nephropathy are two major causes of death of patients with diabetes. Extra generation of reactive oxygen species (ROS), induced by hyperglycemia, is considered as the main reason for the development of these diabetic complications. Transcription factor, NFE2-related factor 2 (Nrf2), is a master regulator of cellular detoxification response and redox status, and also provides a protective action from various oxidative stresses and damages. Recently we have demonstrated its important role in determining the susceptibility of cells or tissues to diabetes-induced oxidative stress and/or damage. Therefore, this review will specifically summarize the information available regarding the effect of Nrf2 on the diabetic complications with a focus on diabetic cardiomyopathy and nephropathy. Given the feature that Nrf2 is easily induced by several compounds, we also discussed the role of different Nrf2 activators in the prevention or therapy of various diabetic complications. These findings suggest that Nrf2 has a potential application in the clinic setting for diabetic patients in the short future.

## 1. Introduction

Generally speaking, diabetic cardiovascular complications include macrovascular disease (e.g., stroke and atherosclerosis) and microvascular disease (e.g., retinopathy and nephropathy) [1]. Diabetic nephropathy as one of microvascular diseases and diabetic cardiomyopathy as one of macrovascular diseases are two common complications of diabetes and also two main causes of the mortality for diabetic patients. The prevention of diabetic nephropathy and cardiomyopathy has become a global concern for those who are working in diabetic care and management. Although glucose control, blood pressure, lipid lowering, and the blockade of the renin-angiotensin system [2] were used for the treatment of diabetic patients, the development and progression of nephropathy and cardiomyopathy in the patients with diabetes remains unpreventable. Therefore, to develop an effective approach to prevent or delay the development and progression of these lethal complications for diabetic patients is urgently needed.

Hyperglycemia, hyperlipidemia and inflammation were three main metabolic abnormalities in diabetes, all which are able to stimulate generation of reactive oxygen or nitrogen species (ROS or RNS). Extra generation of these species is known to be critically causative of the development of diabetic complications, including diabetic nephropathy and cardiomyopathy [3–6]. Therefore, antioxidant prevention or therapy of diabetic complications has been attractive, but to date, there was no any antioxidant that was found to be efficiently applied in clinics [4–6].

**Figure 1:**
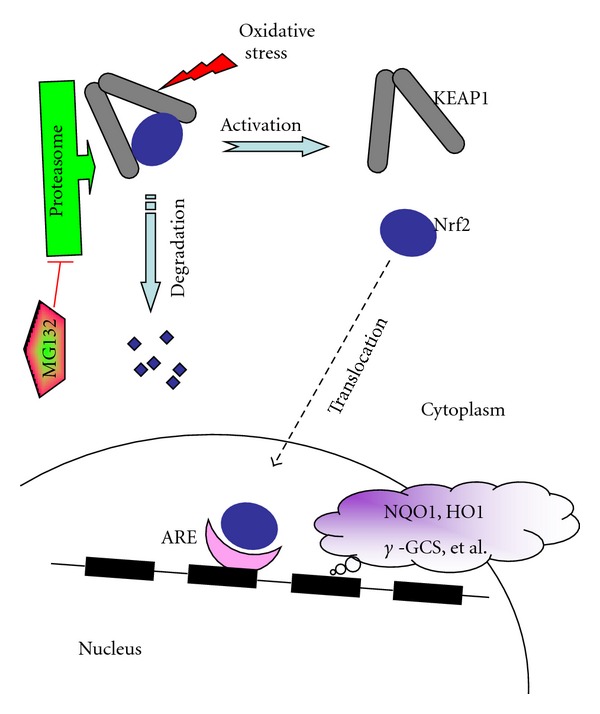
The simplified regulation of Nrf2 by Keap1 and the mechanism of MG132 to activate Nrf2. In general condition, Nrf2 combined with Keap1 in cytoplasm. Under oxidative stress, Nrf2 is free from Keap1 and translocates into nuclear. Through binding with antioxidant response element (ARE), Nrf2 regulates the expression of its downstream target genes to prevent oxidative stress and damage. MG132 could prevent Nrf2 degradation by inhibiting proteasome (adapted from [7, 8]).

The transcription factor NFE2-related factor 2 (Nrf2) as one member of the cap'n'collar family is a master regulator of cellular detoxification responses and redox status [9, 10]. As illustrated in Figure 1, under physiological conditions Nrf2 locates in the cytoplasm and combines with its inhibitor kelch-like ECH-associated protein 1 (KEAP1) [11]. KEAP1 could mediate a rapid ubiquitination and subsequent degradation of Nrf2 by the proteasome [11]. Upon exposure of cells to oxidative stress or electrophilic compounds, Nrf2 is free from KEAP1 and translocates into the nucleus to bind to antioxidant-responsive elements (AREs) in the genes encoding antioxidant enzymes such as NADPH quinone oxidoreductase (NQO1), glutathione S-transferase, heme oxygenase-1 (HO1), and *γ*-glutamylcysteine synthetase, increasing their expression to play a role of detoxification, antioxidation, and anti-inflammation [11, 12].

Recently, several studies have indicated preventive effect of Nrf2 on high glucose- (HG-) induced oxidative damage in the cultured cells and potentially on the diabetic complications in animal models [13]. Although a few good reviews on the general features of Nrf2 in the oxidative stress and damage related to diabetes are available now [14, 15], we would like to briefly review the information in terms of the protective role of Nrf2 in the development of diabetic complications with a specific focus on nephropathy and cardiomyopathy. Given the feature that Nrf2 is easily induced by several compounds, we also discussed the effect of several Nrf2 activators in the prevention or therapy of these two major complications.

## 2. Diabetes Upregulates Nrf2 Expression and Function in the Heart and Kidney

It is known that Nrf2 expression and function in the cells *in vitro* and tissues *in vivo* are increased in response to oxidative stress. Since several studies have indicated that the induction of ROS and/or RNS by HG in the cultured cardiovascular cells and renal cells, whether HG could elevate the Nrf2 expression and activation and its downstream gene expression in these cells has been investigated [13, 16, 17]. Treatment with glucose at 20 and 40 mM for 24 h increased the expression of Nrf2 mRNA in primary cardiomyocytes or H9C2 cardiac cell line. NQO1, a prototype of Nrf2-regulated chemical-detoxification gene, was induced to be overexpressed in cardiomyocytes by such HG exposure too. Immunoblotting confirmed the protein expression and induction of Nrf2 and HO1 in cardiomyocytes. Immunofluorescent confocal microscopic examination of the cells revealed that HG treatment significantly increased the nuclear and total cell staining of Nrf2 in comparison with the control cells, indicating that glucose indeed increased the protein level and nuclear accumulation of Nrf2 [16]. Used human mesangial cells (HRMCs), Jiang et al. also demonstrated HG-induced elevation of nuclear protein level of Nrf2 along with upregulation of the mRNA level of NQO1, HO-1, and GST [17]. To further confirm the notion that Nrf2 activation by HG is through generation of ROS, N-acetylcysteine (NAC), an ROS scavenger, was included in the medium. As expected, NAC inhibited the activation of HG-induced Nrf2 and NQO1. In addition, HG-induced Nrf2 activation in other cells such as endothelial cells [13, 18] and vascular smooth muscle cells [19] was also reported. Collectively, these results indicate that HG or hyperglycemia is able to activate the Nrf2 pathway through generation of ROS.

Upregulation of Nrf2 and/or its downstream antioxidant genes in response to hyperglycemia were found not only in the cultured cells, but also in the heart and kidney of diabetic mice that were also observed. We have used C57BL/6 mice to make type 1 diabetes with a single dose of streptozotocin (STZ). At two weeks after hyperglycemia, we found a significant upregulation of Nrf2 downstream genes NQO1 and HO1 mRNA expression [16]. Jiang et al. have examined if Nrf2 is activated in the kidney of STZ-induced diabetic mice. They used multiple low doses of STZ to induce type 1 diabetes in C57BL/6 mice. At 16 weeks postinjection, Nrf2 expression in the glomeruli of diabetic mice was greatly enhanced and Nrf2 nuclear staining was observed. Activation of Nrf2 was confirmed by upregulation of NQO1 in the glomeruli of diabetic mice [17].

Diabetes-upregulated activation of Nrf2 expression was also observed in the kidney and heart of diabetic patients. In the study by Jiang et al., the diabetic nephropathy kidney tissues were obtained from patients with proteinuria that underwent a renal biopsy for diagnosis of diabetic nephropathy and nondiabetic patients as control. They used these normal and diabetic nephropathy glomeruli to perform immunohistochemical analysis, showing that Nrf2 was hardly expressed in normal glomeruli, whereas it was upregulated in diabetic nephropathy glomeruli. In addition, cells with high expression of Nrf2 in the nucleus were identified as mesangial cells. NQO1 was also activated in glomeruli of the patients with diabetic nephropathy [17]. In contrast with diabetic nephropathy patients, Tan et al. have demonstrated a different finding in terms of Nrf2 expression in the cardiac tissue from diabetic patients and control [20]. Tissue sections of left ventricles were obtained from autopsy heart specimens of humans with or without diabetes (all diabetic males had histories of hypertension and cardiac dysfunction). Nrf2 expression in the nuclei was significantly downregulated compared to control heart. Reasons for the discrepancy between diabetic kidney and heart remain unclear now based on the limited date. However, several possibilities should be kept in mind: (1) case numbers are too small; (2) organ's different responses may be related; (3) tissues from control groups may be an important issue since what these patients were exposed to were unclear; (4) the last is the period of diabetes history.

In support of the last notion listed above, our recent finding demonstrated that Nrf2 protein expression was slightly increased in the heart of mice with two months hyperglycemia but significantly decreased in the heart of mice with 5 months hyperglycemia [20]. Combined our early study [16] in which Nrf2 downstream genes were increased in the heart of diabetic mice at 2 weeks after STZ-induced hyperglycemia, we assume that Nrf2 is adaptively trying to remain functional to overcome diabetic damage at the early stage of diabetes. At the late stage of diabetes, however, cardiac antioxidant function is further impaired, leading to a decrease in cardiac Nrf2 expression. Therefore, these above studies imply the preventive function of Nrf2 against diabetes-induced oxidative damage.

## 3. Downregulation of Nrf2 Gene Accelerates Diabetic Pathological Effect on the Heart and Kidney

To gain insight into the role of Nrf2 in prevention of diabetic complication, Yoh et al. have performed the first study using Nrf2-KO mice [21]. They used STZ to induce diabetes in both Nrf2-KO and their wild-type (WT) C57BL/6 mice and found that compared to WT diabetic mice, Nrf2-KO diabetic mice exhibited a deterioration of renal function gradually over the 10-week observation period, along with urinary excretion of nitric oxide metabolites and the occurrence of 8-nitroguanosine, as index of glomerular lesions, during the early stages after treatment. The increased susceptibility of Nrf2-KO mice to diabetes-induced renal damage was further and systemically examined by Jiang et al. [17]. In this study, they used STZ to induce diabetes with Nrf2-KO and WT mice and demonstrated the following evidence: (1) at 16 weeks post-injection, Nrf2-KO diabetic mice showed higher renal ROS production, greater oxidative DNA damage and renal injury compared with WT diabetic mice; (2) Nrf2-KO diabetic mice had more severe glomerular injury than WT diabetic mice, shown by increased glycogen deposition and severe glomerulosclerosis; (3) Nrf2-KO mice had higher TGF-*β*1 transcription and fibronectin expression, this work clearly indicates a protective role of Nrf2 in diabetic nephropathy; (4) to directly link the Nrf2 to the renal protection from diabetes, particularly hyperglycemia, they used human renal mesangial cells to show that HG-induced significant increase in the expression of several fibrotic mediators, including TGF-*β*1, could be enhanced by knockdown of Nrf2 by siRNA.

In consistence with the renal protection of Nrf2 from diabetes, we demonstrated that HG-induced ROS generation in both primary neonatal and adult cardiomyocytes from the WT mouse heart, whereas, in the cardiomyocytes from Nrf2-KO mice, ROS was significantly higher under basal conditions and HG remarkably further increased ROS production in concentration and time-dependent manners [16]. In addition, HG also induced significantly higher levels of apoptosis at lower concentrations and in shorter time in Nrf2-KO cardiomyocytes than in WT cardiomyocytes. Primary adult cardiomyocytes from control and diabetic mice that was induced by STZ also showed dependence on Nrf2 function for isoproterenol-stimulated contraction [16].

In fact, Nrf2 activation not only protects the kidney and heart from diabetes-induced oxidative damage, but also the other organs from diabetes. For instance, Ungvari et al. have tried to elucidate the homeostatic role of adaptive induction of Nrf2-driven free-radical detoxification mechanisms in endothelial protection under diabetic conditions. They fed both Nrf2-KO and WT mice with high-fat diet (HFD). HFD-induced increases in vascular ROS levels and endothelial dysfunction were significantly greater or more severe in Nrf2-KO mice than WT mice [18].

## 4. Prevention of Diabetic Complications by Activation of Nrf2 with Various Activators

**Figure 2:**
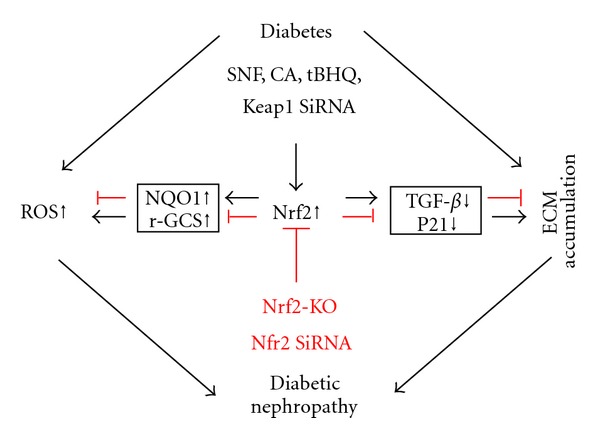
The protection by Nrf2 activation from diabetic nephropathy. Two main pathogenic factors leading to diabetic nephropathy in diabetic patients are increased ROS/RNS and extracellular matrix (ECM) accumulation. Activation of Nrf2 by SNF, CA, tBHQ, and Keap1 SiRNA or activation of Nrf2's downstream targets genes such as NQO1 and r-GCS plays an important role in preventing ROS/RNS-induced damage. At the same time, the expression of TGF-*β* and P21 was inhibited, leading to a prevention of ECM accumulation. Inhibition of Nrf2 in Nrf2-KO animals or by Nfr2 siRNA resulted in an enhancing diabetic effects. This illustration was made mainly based on a published study [22].

The information from the above parts indicates that Nrf2 as an adaptive mechanism is upregulated in the cells exposed to HG or tissues of diabetic animals and patients. Deletion of Nrf2 gene causes a significant increase in the susceptibility of cells or tissues to HG- or diabetes-induced oxidative damage and dysfunction, as illustrated in Figure 2. Therefore, upregulation of Nrf2 expression and function by various approaches may provide a preventive effect on diabetes-induced oxidative damage and consequent complications, to support of which several studies have been done with very multiple beneficial effects on the diabetic complications, as summarized in Table 1.

### 4.1. *In Vitro* Evidence

The first study using Nrf2 activator to prevent HG-induced damage was done by Xue et al. [13]. They used sulforaphane (SNF) to induce nuclear translocation of Nrf2 with significant increases in its downstream antioxidant genes such as three- to five-fold increased expression of transketolase and glutathione reductase [13]. SNF treatment significantly prevented HG: increased the formation of ROS and the activation of the hexosamine and PKC pathways, both which have been well defined as important cellular changes in diabetic effect on the target organs. After this study, several other studies with different Nrf2 activators in different kinds of cells also reported similar preventive effects on HG-induced oxidative damage (see Table 1). For instance, the effects of magnesium lithospermate B (LAB) against HG-induced oxidative stress and damage via activation of Nrf2 have been evaluated in vascular smooth muscle cells [19].

Zheng et al. have systemically examined the preventive effect of Nrf2 activation with its activators on HG-mediated mesangial cell growth inhibition and hypertrophy. They used human mesangial cell line (HRMC) and three kinds of Nrf2 activators, *tert*-Butylhydroquinone (*t*BHQ), SNF, or cinnamic aldehyde (CA). The growth curve for HRMCs growing in HG media was found to be below compared to those either growing in control or in HG with Nrf2 activators. Staining with Ki67 showed that hyperglycemia inhibited cell proliferation, which was counteracted by activation of Nrf2. The size of HRMCs growing in HG was larger than those in control media, and the increased HRMCs' size induced by HG was significantly improved by three Nrf2 activators. Collectively, these results demonstrate that hyperglycemia-induced cell growth inhibition, and that hypertrophy can be attenuated by activation of the Nrf2 pathway. To gain molecular understanding of how Nrf2 activation relieves HRMC hypertrophy and growth inhibition under HG conditions, the expressions of TGF-*β*1 and its downstream effectors were further analyzed. They found that HG-induced upregulation of TGF-*β*1, fibronectin, collagen IV, and p21 in HRMCs was suppressed by treatment with three Nrf2 activators.

### 4.2. *In Vivo* Evidence

The preventive effect of Nrf2 activation on diabetic complications in animal models has been explored recently by several groups with different activators (see Table 1). Resveratrol, a red wine antioxidant and a natural phytoalexin, has already been used in prevention and therapy of cancer [33], regulation of blood platelet functions, and protection of cardiovascular diseases [34]. By treating STZ-induce diabetic rats with resveratrol, Palsamy and Subramanian not only confirmed the protection of resveratrol to diabetic nephropathy but also found that resveratrol could normalize the protein expression of Nrf2, and its downstream genes such as *γ*-GCS and HO-1 in diabetic kidney [31]. Similarly by treating with 1% *t*BHQ in STZ-induce diabetic mice diet, Li et al. also demonstrated that renal expression of Nrf2 and downstream antioxidants increased along with significant decreases in the extracellular matrix deposition and malondialdehyde concentrations in the glomeruli compared to the diabetic mice with regular diet [28].

As mentioned in Section 1, under physiological conditions, Nrf2 locates in the cytoplasm and combines with its inhibitor Keap1 that leads to Nrf2 degradation by the proteasome (Figure 1). It is known that MG132 acts as a proteasome inhibitor, so that it may activate Nrf2 through decreasing the degradation of Nrf2. Luo et al. have first explored the possible prevention of diabetic nephropathy by activation of Nrf2 with MG132 [8]. They treated STZ-induced diabetic rats with MG132 (10 *μ*g/kg) for 12 weeks and they found the following important outcomes. (1) Diabetes-increased 24-h urinary protein excretion rate and renal pathological changes were all improved after MG132 administration. (2) Diabetes-enhanced renal 26S proteasome activity and concentration were effectively reduced after MG132 administration. (3) Diabetes-increased nitrosative damages in kidneys were decreased after MG132 treatment. (4) Renal mRNA and protein expressions of Nrf2 in diabetic rats were upregulated by MG132 compared to diabetes alone. Accordingly, diabetes-decreased renal mRNA expression of superoxide dismutase 1 (SOD1), catalase, and glutathione peroxidase (GPx) was restored after MG132 intervention. (5) Diabetes-increased renal nuclear factor *κ*B (NF-*κ*B) activity was inhibited after MG132 administration. These data suggest that inhibition of the proteasome by MG132 has a preventive effect on the development and progression of diabetic nephropathy in rats through the upregulation of Nrf2-dependent antioxidant genes (Figure 1).

In line with the above findings for the renal protection by MG132 activation of Nrf2, a series of synthetic triterpenoids, CDDO, and CDDO-imidazolide as potent inducers of Nrf2/ARE signaling has been developed and used to prevent several diseases related to inflammatory and oxidative damage [35–38]. Recently, a novel synthetic triterpenoid derivative, dihydro-CDDO-trifluoroethyl amide (dh404), has been developed to activate Nrf2 and suppresses oxidative stress. Dh404 interrupted the Keap1-Cul3-Rbx1 E3 ligase complex-mediated Nrf2 ubiquitination and subsequent degradation saturating the binding capacity of Keap1 to Nrf2, thereby rendering more Nrf2 to be translocated into the nuclei to activate Nrf2-driven gene transcription [39]. We have shown that activation of cardiac Nrf2 by Dh404 in STZ-induced diabetic mice significantly prevented diabetes-induced cardiac oxidative damage and insulin resistance [20]. More importantly, Bardoxolone methyl (aka CDDO-Me or RTA 402) has been clinically explored and extensively discussed for its preventive and therapeutic effects on diabetic nephropathy [40–45].

Although the studies discussed above and others listed in Table 1 have phenomenally suggested the involvement of Nrf2 activation in the preventing diabetic nephropathy, it remains unclear whether upregulated Nrf2 by these activators such as resveratrol, *t*BHQ, or MG132 is really or not the mediator to prevent complications. This uncertain conclusion was solved by a recent systemic study by Zheng et al. The beauty of the Zhang et al. study is that they used two activators of Nrf2: SNF and CA to treat STZ-induce diabetic model in both Nrf2-KO and WT mice [22]. They demonstrated that the activation of Nrf2 and its downstream targets NQO1 and *γ*-GCS could improve metabolic disorder and alleviate renal damage in STZ-induced WT diabetic mice, but not in Nrf2-KO diabetic mice. This study conclusively indicates the requirement of Nrf2 for SNF- and CA-induced renal protection against diabetes [22].

Besides the studies discussed above, several other studies also demonstrated the preventive effects of Nrf2 activation on diabetic complications with different Nrf2 activators, which are summarized in Table 1. It should be noted that not all Nrf2 activators have the same effects on the activation of Nrf2 and its downstream target genes. For instance, AGE-modified bovine albumin (AGE-BSA) could induce Nrf2 nuclear translocation and enhanced mRNA and protein expression of HO-1 and NQO1, but not glutathione peroxidase-1. Treated with AGE-BSA (100 *μ*g/mL for 24 h), bovine aortic endothelial cells exhibited an adaptive endogenous defense against oxidative stress in diabetes [30]. Pentaerithrityl tetranitrate (PETN), although did not activate Nrf2, significantly activated the expression and function of HO-1, which significantly improved endothelial dysfunction in diabetes by reducing oxidative stress [29].

To further address the importance of Nrf2 downstream protective genes in preventing diabetic complications, there was a very important human study. It is known that NQO1, one important Nrf2 downstream protective components, is an important detoxifying enzyme. To address whether the functional variants of NQO1 can reflect the development of diabetic complications, Ramprasath et al. have analyzed the genotypes of 539 type 2 patients and 285 controls in South Indian population. It is found that the functional variants of NQO1 were associated with the development of coronary artery disease in people with type 2 diabetes [46]. There was an study, in which nonobese and hypoinsulinemic C57BL/6-Ins2(Akita) (C57BL/6-Akita) diabetic mice were treated with telmisartan, an angiotensin II type 1 receptor blocker, for 4 weeks. Vehicle-treated C57BL/6-Akita mice exhibited higher renal NAD(P)H oxidase (NOX) and lower renal SOD activity with increased levels of renal superoxide than the C57BL/6-wild-type nondiabetic mice. Interestingly, telmisartan treatment not only reduced NOX activity but also enhanced SOD activity in C57BL/6-Akita mouse kidneys, leading to a reduction of renal superoxide levels. Furthermore, telmisartan-treated C57BL/6-Akita mice increased the renal protein expression of SOD and Nrf2. In parallel with the reduction of renal superoxide levels, a reduction of urinary albumin levels and a normalization of elevated glomerular filtration rate were observed in telmisartan-treated C57BL/6-Akita mice. Finally, treatment of C57BL/6-Akita mice with apocynin, an NOX inhibitor, also increased the renal protein expression of SOD and Nrf2. Collectively, our data suggest that NOX negatively regulates renal SOD, possibly by downregulation of Nrf2, and that telmisartan could upregulate renal SOD by the suppression of NOX and subsequent upregulation of Nrf2, leading to the amelioration of renal oxidative stress and diabetic renal changes [32].

## 5. Conclusions

The prevalence of diabetes dramatically increases in worldwide and its complications significantly affect the life quality of diabetic patients. Diabetic nephropathy and cardiomyopathy are the two major causes for the mortality of diabetic patients. However, there was no an effective approach to prevent the development of these complications for the patients with diabetes. Recently, studies have indicated that Nrf2 as a pivotal mediator for the antioxidant defense system in our body plays a critical role in preventing diabetes-induced oxidative stress/damage, inflammation, and organ's dysfunction. Although there remain many questions to be further investigated, the potential beneficial effects of upregulation of Nrf2 and/or its downstream protective components has attracted the attention of basic scientists and clinical physicians to consider its potential application in the clinic.

## Figures and Tables

**Table 1:** Nrf2 activators were treated to diabetic animals and cells.

Nrf2 activators	Mechanisms	Target organs	Species	Ways and volume	References
Insulin	Nuclear translocation	Brain endothelial cell	Human	100 nM, 30 min	[23]

		HMEC-1 endothelial cell	Human	4 mol/L, 6–48 h	[13]
		Pancreatic islet RIN cells	Mice Rat	40 *μ*g/kg, IP daily, 8 days 10 *μ*M, 3 h	[24]
Sulforaphane	Disrupt the Keap1-Nrf2 complex nuclear translocation	Kidney	Mice	12.5 mg/kg, p.o. 16 weeks	[22]
		Mesangial cells	Human	1.25 mmol/L	
		Nerve Neuro2a cell	Rat	0.5 and 1 mg/kg, 6 weeks 5.5 mM	[25]

Oltipraz	Nuclear translocation	Liver	Mice	150 mg/kg, IP tertian, 5 times	[26]
		Adipose/muscle	Mice	0.75 g/kg p.o., 28 weeks	[27]

tBHQ	Enhance expression and nuclear accumulation	Kidney	Mice	1% p.o., 4 and 12 week	[28]
		Renal mesangial cells	Human	6.25 mmol/L	[22]

MG132	Decrease degradation	Kidney	Rat	10 *μ*g/kg IP, daily, 12 weeks	[8]

PETN	Induce HO-1	Blood vessel	Rat	15 mg/kg/day, p.o. 7 weeks	[29]

LAB	Activate NQO1	Vascular smooth muscle cells Vessel tissue	Rat	50 *μ*mol/L 50 mg/kg IP. daily, 15 days	[19]

AGE-BSA	Nuclear translocation	Aortic endothelial cells	Bovine	100 *μ*g mL^−1^, 0–24 hours	[30]

Resveratrol	Increase expression	Kidney	Rat	5 mg/kg, p.o., 30 days	[31]

DH404	Disrupt the Keap1-Nrf2 complex	HL-1 cells Heart	Mice	200 nmol/L, 12 hour 10 mg/kg, IP., tertian 2 weeks	[20]

CA	Increase the expression	kidney mesangial cells	Mice Human	25/50 mg/kg, p.o., 16 weeks 5 mmol/L	[22]

Telmisartan	Suppression of NAD(P)H oxidase	Kidney	Mice	5 mg/kg, p.o., 4 weeks	[32]

Notes. RIN cells: rat pancreatic *β*-cell line RINm5F; HMEC-1 cells: human microvascular endothelial cells; tBHQ: tert-butylhydroquinone; PETN: pentaerithrityl tetranitrate; LAB: magnesium lithospermate B; AGE-BSA: AGE-modified bovine albumin; CA: cinnamic aldehyde; HL-1 cells: adult murine atrial cardiomyocyte tumor lineage p.o.: diet; IP: intraperitoneal injection.
